# Exogenous Melatonin Mitigates Acid Rain Stress to Tomato Plants through Modulation of Leaf Ultrastructure, Photosynthesis and Antioxidant Potential

**DOI:** 10.3390/molecules23020388

**Published:** 2018-02-11

**Authors:** Biswojit Debnath, Mubasher Hussain, Muhammad Irshad, Sangeeta Mitra, Min Li, Shuang Liu, Dongliang Qiu

**Affiliations:** 1 College of Horticulture, Fujian Agriculture and Forestry University, Fuzhou 350002, China; biswo26765@yahoo.com (B.D.); mubasherhussain05uaf@yahoo.com (M.H.); irshadaup@gmail.com (M.I.); sangeeta.dae@hotmail.com (S.M.); liminzyl@sina.com (M.L.); liushuangsyau@aliyun.com (S.L.); 2 Department of Horticulture, Sylhet Agricultural University, Sylhet 3100, Bangladesh

**Keywords:** simulated acid rain, melatonin, reactive oxygen species, antioxidant activity, *Solanum lycopersicum* L.

## Abstract

Acid rain (AR) is a serious global environmental issue causing physio-morphological changes in plants. Melatonin, as an indoleamine molecule, has been known to mediate many physiological processes in plants under different kinds of environmental stress. However, the role of melatonin in acid rain stress tolerance remains inexpressible. This study investigated the possible role of melatonin on different physiological responses involving reactive oxygen species (ROS) metabolism in tomato plants under simulated acid rain (SAR) stress. SAR stress caused the inhibition of growth, damaged the grana lamella of the chloroplast, photosynthesis, and increased accumulation of ROS and lipid peroxidation in tomato plants. To cope the detrimental effect of SAR stress, plants under SAR condition had increased both enzymatic and nonenzymatic antioxidant substances compared with control plants. But such an increase in the antioxidant activities were incapable of inhibiting the destructive effect of SAR stress. Meanwhile, melatonin treatment increased SAR-stress tolerance by repairing the grana lamella of the chloroplast, improving photosynthesis and antioxidant activities compared with those in SAR-stressed plants. However, these possible effects of melatonin are dependent on concentration. Moreover, our study suggests that 100-μM melatonin treatment improved the SAR-stress tolerance by increasing photosynthesis and ROS scavenging antioxidant activities in tomato plants.

## 1. Introduction

Acid rain (AR) has been considered as a serious environmental issue in Europe, Asia, and North America in recent decades [1,2]. Nowadays, China has become one of the most severely affected regions in the world by acid rain because of rapid economic development [3]. AR mainly developed by two major components of air pollution, namely sulfur dioxide (SO_2_) and nitrogen oxides (NOx) derived from fossil fuel combustion and traffic emission [4]. The indiscriminate and ever-growing practice of energy production might cause extensive degradation of natural resources as well as influenced our life support system [5]. 

In recent years, acid rain deposition also considered as a serious abiotic stress that had a harmful effect on agricultural plants [6,7]. It has been observed that acid rain deposition in the plant caused the ultrastructural changes mainly in the mesophyll, chloroplasts, and mitochondria of the leaf, which resulted in the extreme production of ROS [8,9]. Also, plant exposed to acid rain resulted in characteristic foliar injury symptoms, modified leaf anatomy and necrosis, decrease in the chlorophyll concentration, and altered respiration and photosynthesis [10,11]. Moreover, decreasing of photosynthetic rate and chlorophyll content which hamper the normal plant growth by altering the antioxidant activities because of AR [12]. Furthermore, extensive ranges of physiological changes including the accumulation of reactive oxygen species and lipid peroxide were observed in response to acid rain stress which leads to oxidative damage in a plant cell [1]. On the other hand, plants enhanced antioxidant defense system (both enzymatic and nonenzymatic) to protect themselves against the detrimental effects of ROS under acid rain stress conditions as similar to other environmental stress [1,13]. However, the severity of oxidative damage and breakdown of plant antioxidant defense system occurred if duration and severity of stress are more than the defense capacity of the plant [14].

Melatonin (*N*-acetyl-5-methoxytryptamine) is a low-molecular-weight molecule having an indole ring structure widely present in living organisms extending from bacteria to mammals [15,16]. This molecule, previously known as an animal hormone involved in numerous biological process [17]. In recent year, it has been explicitly confirmed the presence of melatonin in almost all plant organs such as seeds, roots, stems, leaves, flowers and fruit [18]. Different bioactive metabolites of melatonin like 5-methoxytryptamine (5-MT), cyclic 3-hydroxymelatonin (c3OH M), *N*^1^-acetyl-*N*^2^-formyl-5-methoxykynuramine (AFMK), and *N*^1^-acetyl-5-methoxykynuramine (AMK) transformed enzymatically, pseudo-enzymatically, or nonenzymatically to advanced antioxidant capacity [19]. Melatonin and its metabolites are known as both a hydrophilic and a hydrophobic antioxidant because of their easy solubility in water and fat behavior. This is the one of reasons that melatonin and its metabolites considered as the best antioxidant substances. Consequently, it can easily cross the cell membranes and dispense to any aqueous section, such as cytosol, nucleus and mitochondria [16,20].

In plants, a primary function of melatonin is to assist as the first line of defense against internal and environmental oxidative stress and also act as a growth stimulator [16]. Several antioxidant activities of melatonin in plants occurred by directly scavenging free radicals; exciting antioxidant enzymes; enhancing the other antioxidants activities; defending antioxidant enzymes from oxidative damage; or growing the proficiency of the mitochondrial electron transport chain, thereby enabling electron leakage and thus dropping the free radical’s generation [21,22]. Previous research have been confirmed that exogenous melatonin application improved tolerance against alkaline stress [23,24], cold stress [25], drought stress [26], Cd stress [27], Nano-ZnO stress [28], and salinity stress [29] by improving photosynthesis activities, lessens oxidative damage by scavenging ROS and motivating antioxidant systems in plants. 

So far, the impact of exogenous melatonin in acid rain stress has not been testified. To better understand the mechanism of melatonin in simulated acid rain stress responses, we sought to analyze plant growth, ultrastructure of leaves, photosynthesis activity, oxidative and membrane damage, and different antioxidant compounds. We hypothesize that melatonin might have a protective role in simulated acid rain stress tolerance in plants by involving different physiological mechanisms. Given this hypothesis, we carried out a set of experiments in tomato (*Solanum lycopersicum* L. cv. Micro-Tom) as a model plant for other Solanaceae crops. This study explored the possible plant physiological mechanisms of melatonin in acid rain stress response, which might have a potential suggestion for ensuring food safety and food security in marginal agriculture.

## 2. Results

### 2.1. Effects of Melatonin on Growth of Tomato Plant under SAR Stress

**Figure 1:**
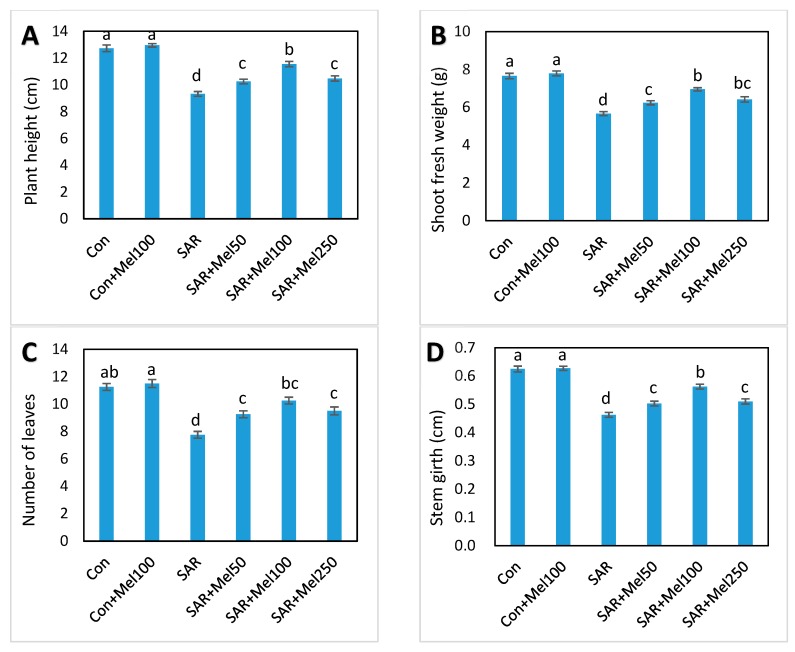
Changes of plant growth parameters in tomato plants by SAR and/or melatonin treatments. (**A**) plant height; (**B**) fresh weight of shoot; (**C**) number of leaves; (**D**) stem girth. Data indicates here the mean of four replications. Vertical bars indicate the standard error. Means not sharing a common letter within the graph are significantly different at *p* ≤ 0.05 according to Turey’s test.

**Figure 2:**
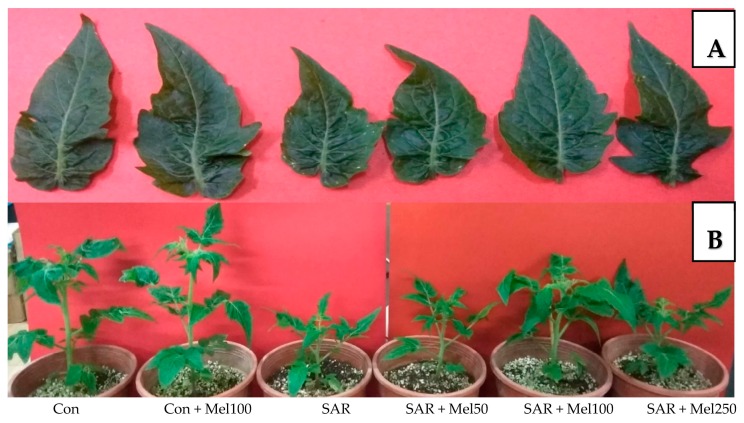
Phenotypic changes of tomato plants in SAR and/or melatonin treatments. (**A**) Leaf phenotype; (**B**) plant phenotype.

To investigate the role of exogenous melatonin in SAR-stress tolerance, first, we observed different phenotypic changes that occurred in tomato plants. As shown in Figure 1, the values of plant height, fresh weight of shoot, number of leaves, and stem girth were reduced by about 27%, 26%, 31% and 27%, respectively, in SAR-stressed plants compared with those in control plants. In addition, plants treated with SAR caused leaf deformation and visible, small white-to-tan dotted spots were observed when compared with control plants (Figure 2). In contrast, the supplementation of 50 μM–250 μM melatonin to SAR-stressed plants showed noticeable lessened these damaging symptoms especially with 100 μM melatonin application (Figure 1 and Figure 2). Whereas, the control plants treated with melatonin had no significant effects on plant growth parameters (Figure 1). These results indicated that exogenous melatonin significantly balanced the lessening in plant growth when exposed to SAR stress.

### 2.2. Effects of Melatonin on Ultrastructure Alterations in Mesophyll Cells and Chloroplast of Tomato Leaf under SAR Stress

**Figure 3:**
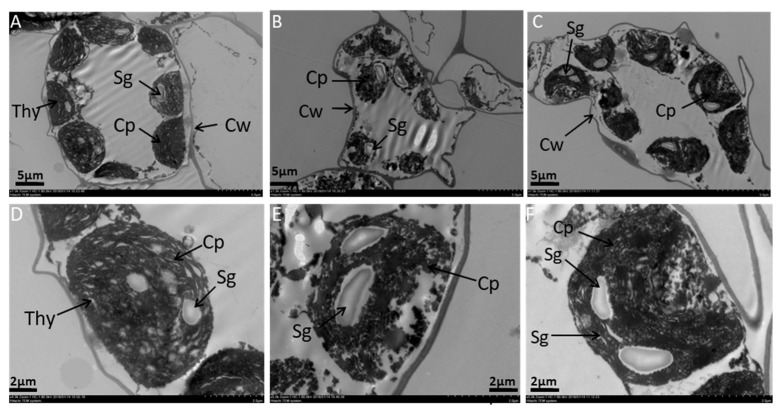
Transmission electron microscopy (TEM) of mesophyll cells of the middle part of the midrib of a leaf, from the top of the plant at the first inflorescence stage. (**A**–**C**) figure shows the TEM structure of whole leaf mesophyll cell of control, SAR, and 100 μM Mel + SAR plants, respectively; (**D**–**F**) figure shows the relatively low magnified view of mesophyll cells of the plant of control, SAR, and 100 μM Mel + SAR plants, respectively. Here, Cp, Cw, Thy, and Sg means chloroplast, cell wall, thylakoids, and starch grana, respectively.

The ultrastructure of whole mesophyll cells and chloroplast of the middle part in the second leaf from the top of the control, SAR, and SAR with 100-μM melatonin-treated plants at the first inflorescence stage showed there were various alterations in the size of the cells and chloroplast structure among these plants’ leaves (Figure 3). The mesophyll cell of SAR-stressed plant’s leaf showed that it had irregular size and shape of chloroplasts, starch grana, and an obvious cell wall without the definite shape of a cell compared with the control (Figure 3A,B). Moreover, the lamellar structure of chloroplasts collapsed, and the shrinking chloroplasts disintegrated with incomplete thylakoid structure were found in the SAR-treated plant’s leaf compared with the control (Figure 3D,E). On the other hand, from the observation of the TEM (transmission electron microscopy) microdiagram of the middle part of the leaf blade of 100-μM melatonin-treated in SAR-stressed plant’s leaf showed that the mesophyll cell of the leaf had approximately equally-sized chloroplasts like the mesophyll cell of the control, regular shape of chloroplasts, was adequately arranged and well-shaped starch grana in chloroplasts, developed thylakoids, visible cell wall, and fewer number of osmophilic plastoglobuli compared with SAR-stressed plant’s leaf (Figure 3). Thus, the leaves of young SAR-stressed plants displayed characteristics of ongoing cell death compared with control plants whereas 100 μM melatonin treatment in SAR-stressed plants showed recovery of cells compared with SAR-alone-treated plants.

### 2.3. Effects of Melatonin on Photosynthesis in Tomato Plant under SAR Stress

**Figure 4:**
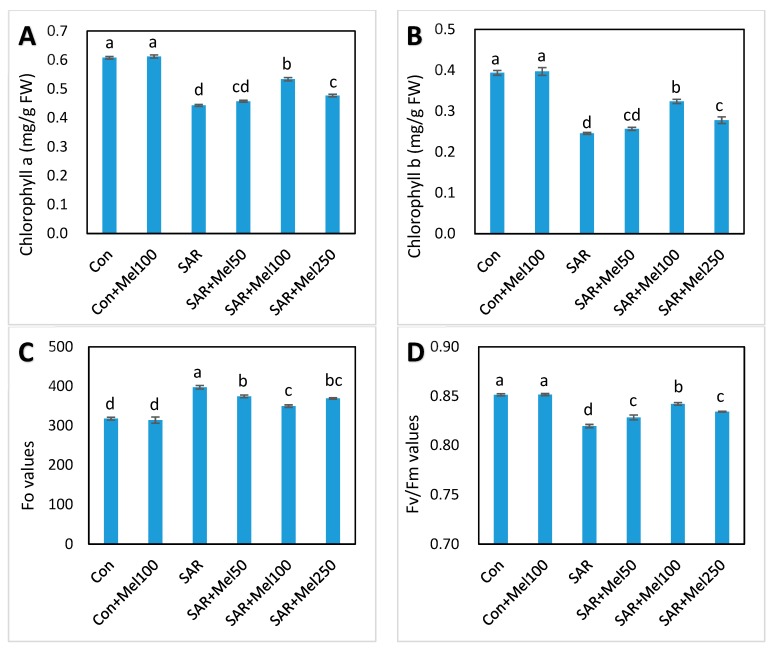
Changes of photosynthesis activities in tomato leaves by SAR and/or melatonin treatments. (**A**) chlorophyll a; (**B**) chlorophyll b; (**C**) Fo values; (**D**) Fv/Fm values. Data indicates here the mean of four replications. Vertical bars indicate the standard error. Means not sharing a common letter within the graph are significantly different at *p* ≤ 0.05 according to Tukey’s test.

To assess the effect of exogenous melatonin on changes of photosynthesis in tomato plants under SAR stress, we observed the concentrations of chlorophyll a and b, and chlorophyll fluorescence in leaves of tomato plants. SAR treatment caused severely decreased chlorophyll a (27.87%), chlorophyll b (35.90%), and Fv/Fm ratio (3.53%) and increased the Fo values (25.02%) compared with those in control (Figure 4). In contrast, almost all treatments of melatonin in SAR-stressed plants significantly reversed the adverse effect of SAR stress (Figure 4). Meanwhile, among the applied concentration of melatonin, 100-μM melatonin treatment in SAR-stressed plant showed the highest recovery in chlorophyll a, chlorophyll b, and Fv/Fm ratio inhibition and the recovered values were 20.46%, 23.08%, and 2.44%, respectively, compared with the SAR-alone treatment (Figure 4). On the other hand, chlorophyll concentration and fluorescence in tomato leaves were not significantly affected by melatonin application under control conditions (Figure 4). These findings suggested that melatonin treatments offset the deterioration in plant photosynthesis associated with SAR stress. 

### 2.4. Effects of Melatonin on H_2_O_2_ and MDA Content under SAR Stress

**Figure 5:**
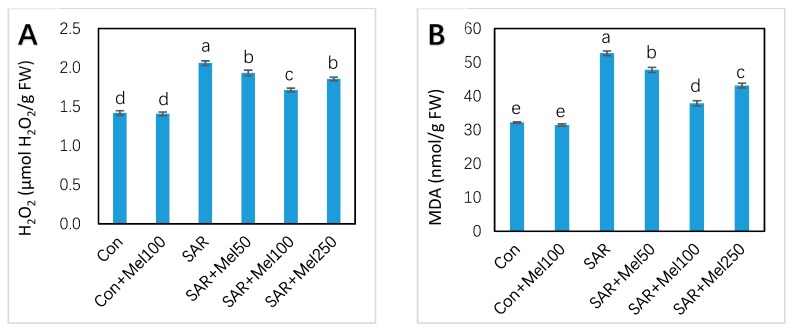
Accumulation of (**A**) H_2_O_2_ and (**B**) MDA concentration in tomato leaves by SAR and/or melatonin treatments. Data indicate here the mean of four replications. Vertical bars indicate the standard error. Means not sharing a common letter within the graph are significantly different at *p* ≤ 0.05 according to Tukey’s test.

To investigate the positive influence of the applied melatonin on SAR-stress tolerance, the H_2_O_2_ concentration acts as an indicator in assessing the ROS scavenging activity, and MDA concentration acts as an indicator in evaluating the membrane damage, were observed in tomato leaves. The results in Figure 5 indicated that SAR treatment caused considerable increased in H_2_O_2_ concentration by 45.07% and MDA concentration by 63.89% in leaves of tomato seedlings compared with the control. However, compared with the SAR-stressed plant, all doses of melatonin treatment showed the comparatively low concentration of H_2_O_2_ and MDA (Figure 5). Meanwhile, the 100-μM melatonin treatment showed the highest decline of H_2_O_2_ (17.01%) and MDA concentration (28.16%) compared with those in SAR-stressed plants (Figure 5). These findings suggested that exogenous melatonin application inhibited the accumulation of H_2_O_2_ and MDA concentration those were produced by SAR stress in tomato plants.

### 2.5. Effect of Melatonin on Enzymatic Antioxidant Compounds under SAR Stress

**Figure 6:**
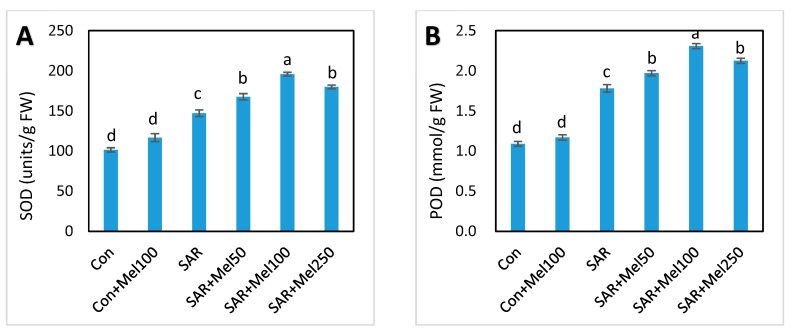

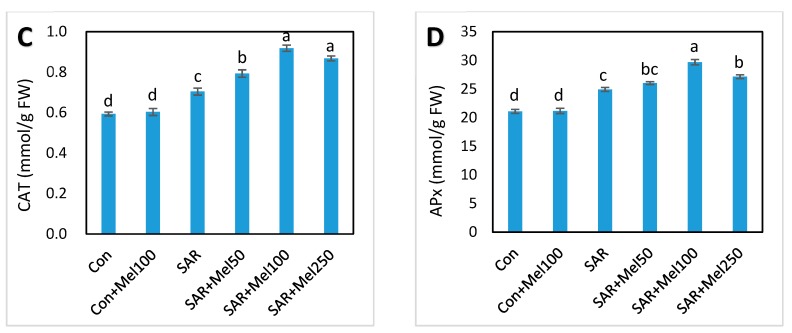
Changes of enzymatic antioxidant activities in tomato leaves by SAR and/or melatonin treatments. (**A**) SOD; (**B**) POD; (**C**) CAT; (**D**) APx. Data indicates here the mean of four replications. Vertical bars indicate the standard error. Means not sharing a common letter within the graph are significantly different at *p* ≤ 0.05 according to Tukey’s test.

To study the role of exogenous melatonin on the enzymatic antioxidative system in tomato plant under SAR-stressed condition, we assessed the activities of superoxide dismutase (SOD), guaiacol peroxidase (G-POD), catalase (CAT) and ascorbate peroxide (APx). In our experiment, SAR treatment increased the activities of antioxidant enzymes in leaves of tomato plants (Figure 6) significantly. Eventually, exogenous melatonin treatment at different concentration in SAR-stressed plants showed further increased of these enzymatic activities at different percentages (Figure 6). In these regards, 100 μM exogenous melatonin showed the highest efficiency and the values of SOD, G-POD, CAT, and APx increased by 33%, 30%, 31%, and 19%, respectively, when compared with SAR-alone treatment (Figure 6). These results indicate that exogenous melatonin encouraged the mitigation of oxidative stress, which was related to the upregulation of enzymatic antioxidant activities in tomato plants.

### 2.6. Effects of Melatonin on Nonenzymatic and Total Antioxidant Activity under SAR Stress

**Figure 7:**
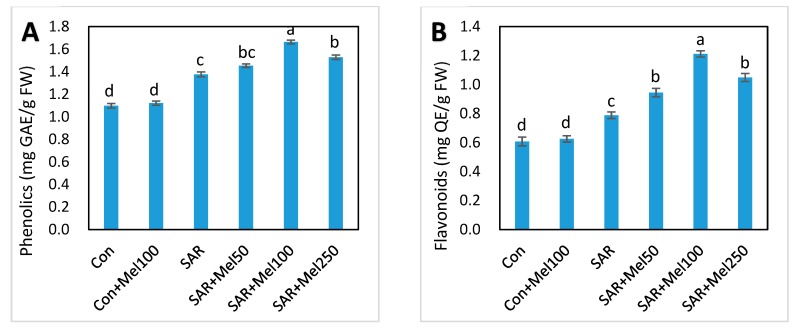

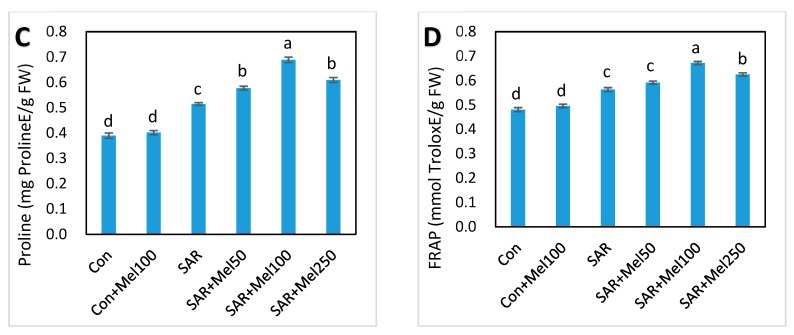
Changes of nonenzymatic and total antioxidant activities in tomato leaves by SAR and/or melatonin treatments. (**A**) phenolics; (**B**) flavonoids; (**C**) proline; (**D**) FRAP (Ferric reducing antioxidant power) values. Data indicates here the mean of four replications. Vertical bars indicate the standard error. Means not sharing a common letter within the graph are significantly different at *p* ≤ 0.05 according to Tukey’s test.

To explore the role of melatonin in nonenzymatic compounds, we estimated total phenolic, flavonoids, and proline content, and verified total antioxidant activity, ferric reducing antioxidant power was calculated in tomato leaves. As shown in Figure 7, total phenolic, flavonoids, proline and total antioxidant content were increased in SAR-stressed plants compared with control plants. Interestingly, the concentration of these nonenzyme compounds and total antioxidant activity further increased at various level by all treatments of melatonin in SAR-stressed plants compared with those in the SAR-alone treatment (Figure 7). Among these results, 100-μM melatonin treatment in SAR-stressed plants showed the best productivity in total phenolic (50.91%), flavonoids (98.36%), proline (71.79%) and total antioxidant activity (39.58%) compared with control plants (Figure 7). These findings provided the hint that exogenous melatonin might have a great role in the alleviation of oxidative stress, which may relate to the stimulation of nonenzymatic antioxidant activities in tomato plants.

## 3. Discussion

Acid rain is one of the major abiotic stresses limiting plant growth and biomass production [7,30]. Growth inhibition is a critical symptom of plants exposed to abiotic stresses. We found that all growth parameters were declined significantly in simulated acid-rain-stressed plants compared with control plants (Figure 1). These consequences may be due to the disruption of chloroplast and lower water potential in the plant cells which turned stomatal closure and limited CO_2_ assimilation, and finally, inhibition of cell division was found in SAR stress condition [8]. In this study, the severity of acid rain-induced growth inhibition in tomato seedlings was lessened when melatonin applied to stressed plants. Moreover, melatonin-treated stressed plants recovered more quickly than untreated stressed plants (Figure 1). The current study agreed with findings of the previous results that spraying of melatonin enhanced plant stress tolerance in field conditions where melatonin act as a possible alleviator of plant growth and development under stress [24,31,32].

Furthermore, in this study, mesophyll cells, and chloroplast distortion were markedly observed in SAR-stressed plant, but these damages were almost mitigated when supplement with melatonin treatment in SAR-stressed plant (Figure 3). Previous study also reported that melatonin-treated plants had less chloroplast damage and thicker leaf tissues and cells compared with stressed plants [33].

Photosynthesis is a basic physiochemical process to maintain plant life activities from which the synthesis of organic compounds of plants occurred through uses of light energy [34]. Acid rain is considered as a serious environmental stress due to its unfavorable effect on bioenergetic processes of photosynthesis [30,35]. The efficiency of photosynthesis depends on chlorophyll content and therefore, reactive oxygen species caused significant decline in the chlorophyll content in the plant due to its fragile nature [16]. So, survival, growth, and production of plants largely depends on functional chlorophyll preservation. The results of our present study showed that the chlorophyll concentration significantly decreased in tomato leaves by simulated acid rain (Figure 4A,B). However, melatonin-treated plants had higher chlorophyll concentrations than the untreated plants (Figure 4A,B), suggesting that melatonin might have protective effects on chlorophyll concentration against acid rain stress in tomato leaves. Meanwhile, the alleviation of chlorophyll degradation was also found in previous research by exogenous melatonin application to improvement in photosynthesis of plants under different stresses [26,36].

On the other hand, chlorophyll fluorescence analysis is a quick, influential and consistent technique to observe photosynthesis in any stressed plant [37]. The primary photosystem II (PSII) of photosynthetic apparatus (Fo and Fo/Fm value) plays a significant role in the conversion of energy process and is a good indicator of acid rain stress in plants [7,11]. Therefore, our results showed that the fluorescence level, when the plastoquinone electron acceptor pool (Qa) is fully oxidized (Fo value), increased and the maximum quantum efficiency of photosystem II (Fv/Fm ratio) declined under SAR stress in tomato seedlings. However, Fo values decreased and the Fo/Fm ratio increased in melatonin-treated stressed plants compared to untreated stressed plants (Figure 4C,D). Similarly, several studies have confirmed that melatonin can boost the photochemical efficiency of PSII photosynthetic apparatus [26,28].

Hydrogen peroxide is one of the most active, toxic and destructive ROS, and more stable than others [38]. The accretion of free radicals and cellular damage destroyed the balance between the formation and accumulation of ROS. Findings of previous studies showed that acid rain stress causes ROS accumulation and breaks down the balance between ROS generation and detoxification [1,39]. Our results showed that SAR stress increased the production of H_2_O_2_ in tomato leaves, but this phenomenon was markedly decreased by the application of melatonin to SAR-stressed plants (Figure 5A). Similar assumptions have been described under oxidative stress in plants by previous research [40].

On the other hand, membrane damage is a good indicator to measure the level of lipid destruction under different environmental stresses. Plant exposed to acid rain increased H_2_O_2_ concentration, which leads to lipid peroxidation and finally causes membrane damage [1,7]. We observed that SAR stress significantly encouraged the accumulation of MDA (Figure 5B). However, melatonin application with SAR treatment in plants significantly reduced SAR-induced MDA levels (Figure 5B). The similar consequence was found in other investigation under different environmental stress condition [25,29]. The reduction of H_2_O_2_ and MDA suggest that exogenous melatonin might have the protective effect on oxidative damage under acid rain stress conditions.

Activities of many antioxidant enzymes and nonenzymes are enhanced in plants to eliminate ROS when plants are subjected to stress [41,42]. With the agreement of other results [13,39], our findings showed that different enzymatic antioxidant compounds such as SOD, POD, CAT, and APx and nonenzymatic antioxidant compounds such as phenolics, flavonoids, and proline were increased to protect against ROS damage by SAR treatment (Figure 6 and Figure 7A–C). In addition, it was observed that exogenous melatonin application in SAR-stressed plants serving as a signal molecule by further increasing these ROS-scavenging antioxidant compounds to enhance defense system compared with SAR-alone treated plants, resulting in the mitigation of oxidative damage (Figure 6 and Figure 7A–C). Moreover, the findings our experiments showed that the activities of these enzymes and nonenzymes enhanced by melatonin treatment in SAR-stressed plants could confer SAR-stress tolerance in tomato plants to some extent. These consequences supported the concept that melatonin may have a vital role in protecting the plants from any abiotic stress conditions and in overcoming damage from oxidative stress at the plant’s cellular level [23,25]. 

In the current study, the total antioxidant activities were determined by ferric-reducing antioxidant power (FRAP) in the leaves of the tomato plant. It was observed that the total antioxidant activities of the plant was partly associated to the ferric-reducing capacity of the bioactive compound [43], and the free radical scavenging activities of phenolics and flavonoids might be related to the reducing antioxidant capacity of plants [44]. Our results showed that the ferric reducing antioxidant activity increased by all doses of melatonin treatment in SAR-stressed plants compared with those in the SAR-alone treatment (Figure 7D). Meanwhile, in agreement with other results [33], current findings evident that melatonin boosted the SAR-stresst tolerance of plants by inducing their antioxidant activities.

In conclusion, melatonin treatment considerably mitigated the growth, photosynthesis, leaf ultrastructure and antioxidant activities, and reduced hydrogen peroxide and lipid peroxide, which leads to SAR tolerance in tomato plants. Among different doses of melatonin treatment in SAR-stressed plants, the 100-μM melatonin treatment showed the marked alleviation by encouraging SAR-stress tolerance in tomato plants. Therefore, this observation can be concerned with emerging new approaches to producing safe food in peripheral areas where acid rain is a limiting factor for crop production.

## 4. Materials and Methods

### 4.1. Plant Materials and Treatments

The experiment was conducted in a greenhouse of Horticulture College, Fujian Agriculture and Forestry University (latitude 26°5′16′′ N, longitude 119°14′6′′ E, altitude 42.09 m), Fuzhou, China, where the temperature, mean relative humidity, light intensity, and photoperiod were 20–25 °C/14–18 °C (day/night), 80%, 150 mol m^−2^·s^−1^ photosynthetically active radiation (PAR), and 15/9 h (day/night), respectively. Tomato seeds (*Solanum lycopersicum* L. cv. Micro-Tom) (lot 5036225409, Ball Horticultural Company, West Chicago, IL, USA) were germinated in a growth substrate containing a mixture of vermiculite, peat, and perlite (1:2:1, *v*/*v*) in a greenhouse. At the three true-leaf stage, uniform seedlings were transferred into a greenhouse pot (24 cm × 14 cm × 15 cm) filled with vermiculite, cover soil and perlite (1:1:1). 

To investigate the role of melatonin in leaf ultrastructure, photosynthesis and antioxidant activities against SAR stress condition, leaves of the tomato plant at the fifth true-leaf initiation stage treated with SAR at pH value of 2.5 while 5.6 was used as a control for every second day at 4:00 p.m. (based on our previous experiment, unpublished data). Sulfuric acid and nitric acid were selected for the preparation of simulated acid rain. Firstly, we prepared solution of sulfuric acid and nitric acid on the mole ratio of 5:1. Then, this acid solution was diluted with distilled water during preparation of SAR with pH 5.6 and pH 2.5, and pH values of SAR were determined by pH meter (Orion 828 acidity analyzer, Chelmsford, MA, USA). Leaves were carefully wetted by spraying of 20 mL SAR at each application. To observe the impact of melatonin in control and stressed plants, 100-μM melatonin was sprayed in control plants and 50, 100, and 250 μM melatonin (PCode: 332675009, Sigma-Aldrich Co., St. Louis, MO, USA) were sprayed in SAR-stressed plants (based on our trial experiment) for every three days. For each treatment, four replications were used, and a bulk of 12 plants represented each biological replicate. Seedlings were treated for 17 days. The second to fourth leaves from the top of the seedlings were used for different analysis. 

### 4.2. Measurement of Plant Growth 

To detect the active change of plant growth, plant height was measured by using a steel ruler. Digital Vernier Calipers were used to measure the diameter of the plant stem, fresh weight of shoot measured by digital weight scale (0.0001–200 g; Shanghai Yoke Instrument Co., Ltd., Shanghai, China).

### 4.3. Observation of Leaf Ultrastructure

To observe the changes of mesophyll cell and chloroplast of a leaf in different treatments condition, the ultrastructure of a leaf was observed by transmission electron microscope (Hitachi, Tokyo, Japan). The middle part of fresh leaves were cut in very small piece (about 1 mm^2^) to ensure uniformity of samples, and these fragments of leaf were used for transmission electron microscopy in accordance to the previously described method [45].

### 4.4. Determination of Photosynthesis Activities

Fresh leaf samples were homogenized with 80% acetone for determination of chlorophyll a and b content in tomato seedlings. Chlorophyll a and b contents were estimated by measuring the absorbance in spectrophotometer at 645 and 663 nm and calculated according to the previously described [46] formulae:

Chlorophyll a (mg/g leaf fresh weight) = [12.7(OD_663_) − 2.69 (OD_645_)] × V/1000 × W (1)

Chlorophyll b (mg/g leaf fresh weight) = [22.9(OD_645_) − 4.68 (OD_663_)] × V/1000 × W (2)

where, OD = optical density, V = volume of sample, W = weight of sample.

Chlorophyll fluorescence was measured using a plant efficiency analyzer (Handy PEA, Hansatech, Norfolk, UK). The measurements were performed on leaves from plants that had been dark-adapted for 30 min before data collection. Fluorescence was induced with a saturating PPFD of 3000 μmol m^−2^·s^−1^ provided by an array of six LEDs (peak at 650 nm). Minimal fluorescence level when plastoquinone electron acceptor pool (Qa) was fully oxidized (Fo value) and maximum quantum efficiency of photosystem II (Fv/Fm ratio) were determined in the second fully expanded leaves from the plant’s top. Fv/Fm value was calculated as (Fm − Fo)/Fm [47].

### 4.5. Assay of Hydrogen Peroxide (H_2_O_2_) and Lipid Peroxide (MDA) Content

For H_2_O_2_, a leaf sample was taken from each of the plants, and treatment was homogenized in liquid nitrogen with trichloro acetic acid (TCA). Homogenates were centrifuged at 12,000 × *g* for 15 min at 4 °C and the supernatant (0.5 mL) was mixed with 0.5 mL of 10 mM potassium phosphate buffer (pH 7.0) and 1 M potassium (1 mL) iodide solution and incubated that mixer by shaker for 5 min at 20 °C. The absorbance of oxidation product formed was estimated in spectrophotometer at 390 nm [48]. The H_2_O_2_ concentration was calculated from the standard curve made with known concentrations of H_2_O_2_ and expressed as μmol·g^−1^ fresh weight (FW) of leaves.

Malondialdehyde content (MDA) was determined by the thiobarbituric acid (TBA) test, which determines the MDA as an end product of lipid peroxidation, was used for the measurement of lipid peroxidation in leaves. MDA was determined according to previously described method [49]. 

### 4.6. Estimation of Enzymatic Antioxidant Compounds

For determination of enzymatic antioxidant activities, a fresh leaf sample (0.2 g) was homogenized in 5 mL of 50 mM potassium phosphate buffer (pH 7.8). The homogenate was centrifuged at 12,000× *g* for 20 min at 4 °C, and the supernatant was collected and preserved it at 4 °C for enzymatic assay. This supernatant was used to measure the activities of superoxide dismutase activity (SOD), guaiacol peroxidase (G-POD), catalase activity (CAT) and ascorbate peroxide activity (APx). The SOD activity was determined by measuring its ability to inhibit photochemical reduction of nitro blue tetrazolium (NBT) in spectrophotometer at 560 nm as previously described [50]. G-POD activity was assessed using guaiacol as a substrate in spectrophotometer at 470 nm [51]. The activity of catalase was calculated in spectrophotometer at 240 nm according to the method as described previously [51]. APx activity in the tomato leaves was determined in spectrophotometer at 290 nm as described by Nakano and Asada [52]. 

### 4.7. Determination of Nonenzymatic Antioxidant Compounds

For estimation of total phenolic and flavonoids, leaf sample (0.2 g) was homogenized with 6 mL of 80% ethanol. Then the ethanolic extract was centrifuged at 12,000× *g* for 20 min at 4 °C and the supernatant was used for the assessment of the total phenolic and flavonoid.

The total phenolics concentration was measured by Folin–Ciocalteu colorimetric method with little modification [53]. Folin–Ciocalteu reagent and 7.5% sodium carbonate were used to develop blue color solution. The absorbance of the developed blue color solution was read at 765 nm after water bath at 30 °C for 90 min. Total phenolic content was expressed as gallic acid standard equivalent (mg) on a fresh weight basis (mg GAE·g^−1^ FW).

Flavonoids were estimated using AlCl_3_ method [54] with quercetin as standard. The absorbance solutions were made by using 5% NaNO_2_, 10% AlCl_3_ and 1 M NaOH. The absorbance was measured against blank at 510 nm using the spectrophotometer. The result was expressed as mg quercetin equivalent per g fresh weight of leaves.

The proline content was estimated following the previously observed methods [55]. Fresh leaf sample was homogenized with 3% aqueous sulfosalicylic acid. The reaction mixture was extracted with acid ninhydrin, glacial acetic acid and toluene. The absorbance read in spectrophotometer at 520 nm and toluene used as a blank. The proline concentration was calculated from a standard curve plotted with l-proline standard solution. 

The antioxidant capacity was estimated according to the ferric-reducing antioxidant power (FRAP) assay [56]. FRAP method counts on the reduction of TPTZ (2,4,6-tri-pyridyl-*s*-triazine)-Fe^3+^ complex to TPTZ-Fe^2+^ form, with a concentrated blue color and absorption maximum at 593 nm. The fresh FRAP reagent was prepared by mixing 300 mM acetate buffer (sodium acetate with glacial acetic and distilled water) at pH 3.6 with 20 mM of FeCl_3_ (dissolved in water) and 10 mM of TPTZ (dissolved in 40 mM of HCl) in the ratio of 10:1:1. The FRAP solution was mixed with the ethanoic leaf extracts and the absorbance was measured at 593 nm in spectrophotometer. FRAP values were expressed as mg Trolox (6-hydroxy-2,5,7,8-tetramethylchroman-2 carboxylic acid) equivalents (Trolox) in 1 g fresh weight of leaves.

### 4.8. Statistical Analysis

The data were subjected to one-way analysis of variance (ANOVA) and general linear method using the SPSS 22.0 for windows statistical package (SPSS, Chicago, IL, USA). Differences between treatments were detected and mean values were compared by Tukey’s test (*p* > 0.05). Each treatment value was the average of four replicates.
